# Floral Traits Predict Frequency of Defecation on Flowers by Foraging Bumble Bees

**DOI:** 10.1093/jisesa/iez091

**Published:** 2019-09-18

**Authors:** Jonah M Bodden, Jenny A Hazlehurst, Erin E Wilson Rankin

**Affiliations:** 1 Department of Entomology, University of California, Riverside, Riverside, CA; 2 Department of Biological Sciences, California State University East Bay, Hayward, CA

**Keywords:** pollinator, pathogen, disease ecology

## Abstract

Flowers may become inoculated with pathogens that can infect bees and other critical pollinators, but the mechanisms of inoculation remain unclear. During foraging, bees may regurgitate or defecate directly onto flower parts, which could inoculate flowers with pollinator pathogens and lead to subsequent disease transmission to floral visitors. We tested if captive eastern bumble bees (*Bombus impatiens*) (Cresson) (Hymenoptera: Apidae) defecate on floral surfaces during foraging and if flower shape played a role in the probability of defecation and the quantity of feces deposited on floral surfaces. Captive *Bombus impatiens* were fed a solution of fluorescent dye and sucrose, then allowed to forage freely on flowers of a variety of shapes in a flight cage. Flowers were then examined under ultraviolet light for fluorescing fecal matter. We found that bumble bees did defecate on floral surfaces during foraging and that composite flowers with a large area of disk flowers were the most likely to have feces on them. Our results point to defecation by bumble bees during foraging as a potential mechanism for inoculation of flowers with pollinator pathogens and suggest that flower shape could play a significant role in inoculation.

Insect pollinators play a key role in sustaining biodiversity and crop yields; a decline in insect pollinator populations could negatively affect ecosystem services and global food production (Aizen et al. 2009). Unfortunately, many insects, including pollinators, are facing global declines (Sanchez-Bayo and Wyckhuys 2019). Insect pollinators are increasingly threatened by disease, in addition to other anthropogenic factors such as climate change, habitat loss, pesticides, and invasive species (Potts et al. 2010, Goulson et al. 2015, O’Neal et al.^2019^). In recent years, we have seen an increase in the spread of infectious diseases in managed pollinators. Pathogen spillover from populations of managed bees may expose wild insect populations to a diversity of virulent diseases against which they are poorly defended (Alger et al. 2019, Loope et al. 2019). Thus, it becomes critical to understand how pathogens can spread through wild pollinator populations.

Increasingly, research efforts have aimed to identify mechanisms of pathogen transmission among pollinators and floral visitors (Folly et al. 2017). Floral traits such as shape and attractiveness can factor into the transmission of plant pathogens by pollinators (McArt et al. 2014) and on the transmission of pollinator pathogens at flowers (McArt et al. 2014, Graystock et al. 2015, Adler et al. 2018). Durrer and Schmid-Hempel (1994) and Graystock et al. (2015) demonstrated that several bee pathogens can be dispersed from an inoculated flower to other flowers by bees, where they may infect other pollinators. Several bee pathogens can be transmitted among bees via the fecal to oral route, including trypanosome parasites (Brown et al. 2003, Gegear et al. 2006), numerous viruses (Ribière et al. 2007, Daughenbaugh et al. 2015), and parasitic microsporidia (Bailey 1955). Although both intraspecific and interspecific pathogen transmission has been observed via shared floral resources, the underlying transmission processes remain largely unresolved. A possible mechanism for the inoculation of flowers with bee pathogens may be via defecation by bees on flowers during resource collection. Individual bee foraging behavior can also be modified by infection, e.g., Figueroa et al. (2019) found that bees infected with the gut pathogen, *Crithidia*, actually defecate more frequently on floral structures. Additional factors may further influence the probability of defecation and hence pathogen transmission, such as individual foraging behavior and flower shape (Adler et al. 2018).

In this study, we looked for evidence of bumble bee defecation on flowers during foraging and analyzed the role of flower shape in the probability and quantity of feces deposited on the flower surface. We fed a solution of sugar and fluorescent dye to Eastern bumble bees, *Bombus impatiens* Cresson (Hymenoptera: Apidae), and invited them to forage on flowers in a flight cage. We hypothesized that during foraging, *B. impatiens* would 1) defecate on floral surfaces and 2) that flower shape (cup, tube, small composite, or large composite) would affect the probability of feces being on the flower. We use the results of these experiments to discuss the potential of defecation during foraging as a mechanism for the inoculation of flowers with bee pathogens.

## Materials and Methods

A colony of *B. impatiens* was attached to a flight cage measuring 1.2 m × 1.2 m × 0.76 m during 4-h foraging trials (Supp Fig. S1 [online only]). We conducted 31 trials between 10 July 2017 and 1 September 2017 inside of a temperature-controlled insectary. Bees were provided with a mixture of 30% sucrose solution and fluorescent dye powder in feeders inside the hive for 12 h prior to each foraging trial. Only one colony was used at a time for each trial. In total, three colonies were used over the course of these experiments. In each trial, 12 individual flowers or inflorescences (depending on the species) were arranged inside the flight cage with flower shapes randomized in a uniform grid (Supp Fig. S1 [online only]). Bees were then released into the flight cage and allowed to forage on the floral array for 4 h per trial. We defined four categories of flower shape: cup (open, uplifted petals), tube (tubular corolla), small composite flower (the diameter of the disk is less than ray petal length), and large composite flower (the diameter of the disk is greater than the ray petal length). The following flower species and flower shapes were used: *Abutilon palmeri* (Malvaceae; Gray) (cup), *Bellis perennis* (Asteraceae; L.) (small composite), *Coreopsis maritima* (Hook. f.) (Asteraceae) (small composite), *Erigeron glaucus* (Asteraceae; Ker Gawl.) (large composite), *Lantana montevidensis* (Verbenaceae; Briq.) (tube), *Salvia sonomensis* (Lamiaceae; Greene) (tube), *Sidalcea malviflora* (Malvaceae; Grey) (cup), and *Sphaeralcea ambigua* (Gray) (cup). All plants were raised in a positive pressure greenhouse. The type and number of each species used varied for each trial based on natural variability in flower availability; however, each trial contained at least one individual of each flower shape (cup, small composite, large composite, tube). Flowers were cut and stems put into flower vials immediately prior to each experiment and emptied of naturally occurring nectar using a micropipette (25 µl; Kearns and Inouye 1993). We then added 100 µl of 30% (wt/wt) sucrose solution to each flower to standardize the reward received by bees at each type of flower, allowing us to focus on the effect of flower shape. In natural environments, flowers often grow densely packed together in space, which contrasts with the individual flowers offered to bees in the foraging arena. To account for this scenario, we added a paper disk 15 cm in diameter approximately 1 cm below the base of each flower in the foraging arena, so as to catch any bee feces that fell near, but not directly on, the flowers in the array, as might happen in a natural context with dense flowers. During each trial, we recorded the total number of workers in the flight cage at 15-min intervals. At the end of each trial, flowers and paper disks were removed from the flight cage and observed under an ultraviolet light to observe any feces.

### Analysis

All statistical tests were done using R version 3.5.2 (R Core Team 2018). To determine whether flower shape had a significant effect on the probability of defecation, we used a binomial logistic mixed model in the package ‘lme4’ (Bates et al. 2015) with the presence or absence of feces (on flower parts and paper disks) as the response variable, the flower shape (cup, tube, large composite, and small composite) as the predictor variable, trial ID as a random effect, and the number of flowers of each shape per trial as an offset variable. We tested this base model with bumble bee colony ID and the mean number of workers observed in the flight cage per trial as random effects and used the function ‘model.sel’ from the package ‘MuMIn’ (Barton 2018) to select the best model. Post hoc Tukey’s tests were conducted in the package ‘emmeans’ (Lenth 2018). To isolate the effect of the ‘flower shape’ factor, we compared the best model from model selection with and without ‘flower shape’ using the anova function in the ‘stats’ package (R Core Team 2018).

## Results

We conducted 31 trials and recorded 28 total fecal events on flowers or paper disks, an average of 0.9 ± 1.0 (SD) feces per trial. For trials where bees did defecate, 54% of all fecal deposits occurred on paper disks, whereas 46% occurred on flower parts. Flower shape influenced the likelihood of fecal presence. Composite flowers with a large disk flower area and smaller ray petal length (defined as ‘large composite’) had a significantly higher probability of fecal presence (*n* = 208 flowers, *z* = 3.83, *P* < 0.001; Supp Tables S1–S3 [online only]; Supp Fig. 2 [online only]). An ANOVA comparing the full model with and without flower shape showed a significant effect of flower shape on the probability of feces (df = 8, χ ^2^ = 17.70, *P* < 0.001; Table 1). Pairwise post hoc Tukey’s tests (Supp Table 3 [online only]) revealed there was a significant difference between large composite flowers and cup flowers (*P* < 0.001), between large and small composite flowers (*P* < 0.05), and between large composite and tubular flowers (*P* < 0.05).

**Table 1.** Results of an ANOVA comparing the full GLMM on the presence or absence of bee feces on or near flower parts with and without the factor ‘flower shape’

Model	df	AIC	LogLik	*P*
Model 1: bin ~ colony + mean.num.workers + offset(log(num.flwers.type)) + (1|trial.id)	5	177.65	−83.82	NA
Model 2: bin ~ flower.shape + colony + mean.num.workers + offset(log(num.flwers.type)) + (1|trial.id)	8	165.95	−74.97	<0.001

*n* = 208 flowers and 31 trials; small composite = flower shape, composite flowers with a shorter disk flower receptacle diameter than ray petal length; large composite = flower shape, composite flowers with a longer disk flower receptacle diameter than ray petal length; tubular = flower shape, flowers with a long tubular corolla; colony = unique colony ID (1–3). AIC (Akaike information criterion); NA (not applicable).

## Discussion

Our results demonstrate that *B. impatiens* predictably defecate on certain flowers during foraging. These findings expand upon the study by Figueroa et al. (2019) by examining defecation patterns on three additional floral shapes, providing a potential mechanism for the inoculation of flowers with bee pathogens that has been observed in other studies (Graystock et al. 2015). The results from these experiments suggest that flower shape has a significant effect on the likelihood of feces being deposited on the flower. This may be in large part due to the fact that floral morphology is known to influence the total time spent on the flower by a visitor (Zung et al. 2015). The longer a visitor spends on a flower, the greater the likelihood of the visitor defecating. Time spent in floral visits by bumble bees can be divided into handling time and extraction time (Inouye 1980). Several floral traits can influence the handling time of flowers by bumble bees, including corolla depth (Inouye 1980), nectar concentration (Klumpers et al. 2019), or whether the floral visitor is collecting pollen versus nectar (Stanley et al. 2017). Extraction time is dependent on the volume of nectar in a flower (Hodges and Wolf 1981) and its viscosity, which is related to the concentration of sugars in nectar (Harder 1986). Composite flowers may have had a higher likelihood of fecal deposition compared with cup- and tube-shaped flowers because they have many small florets per flower, which may require more handling time for bees to forage on as they must move from floret to floret. These factors may also subsequently influence both whether a new defecation event occurs and likelihood of being exposed to previously deposited fecal material.

Where the fecal material is deposited affect the likelihood of whether later foragers will come into contact. Figueroa et al. (2019) showed that defecation on floral structures such as bracts may affect pathogen dispersal and subsequent infection of bees with gut pathogens, such as *Crithidia*. We may further hypothesize that the risk of pathogen transmission would be highest on floral morphologies that have both a high rate of fecal deposition and a high rate of contact by floral visitors. Future studies could explicitly test this and examine if transmission rates are influenced by both fecal deposition and the distribution of floral shapes with a high probability of fecal deposition in the environment. One caveat of our finding that the ‘large composite’ type flower was significantly more likely to be defecated on is that we only had access to one species of plant, *Erigeron*, representing this shape in our trials. Although it is possible that our finding is due to some other unique trait of flowers in the genus *Erigeron* other than shape, it is most likely a combination of the shape and size. All plants used in this study provided floral resources to bees and are visited by bees in both controlled and wild conditions. It is therefore important that future studies also consider additional interspecific variation in flowers that might affect pathogen transmission via handling time or overall preference. Future studies should quantify defecation rates on flowers in the field and quantify rates of inoculations of flowers with pathogens by bee fecal deposition. This study opens the door to future research into the mechanisms whereby flowers may become inoculated with pollinator pathogens and suggests that floral shape may play a significant role in the pathogen transmission process.

## Supplementary Material

iez091_suppl_Supplementary_Figures_and_Tables
