# Supplementary material for: Floral Traits Predict Frequency of Defecation on Flowers by Foraging Bumble Bees
Source: J Insect Sci. 2019 Sep 18;19(5):2. doi: 10.1093/jisesa/iez091 (PMC6937503; doi:10.1093/jisesa/iez091)
Supplement: iez091_suppl_Supplementary_Figures_and_Tables [file iez091_suppl_supplementary_figures_and_tables.docx]

**Supplementary Material**

**Supp. Figure S1.**

Diagram of flight cage showing floral array and bumble bee hive.

**
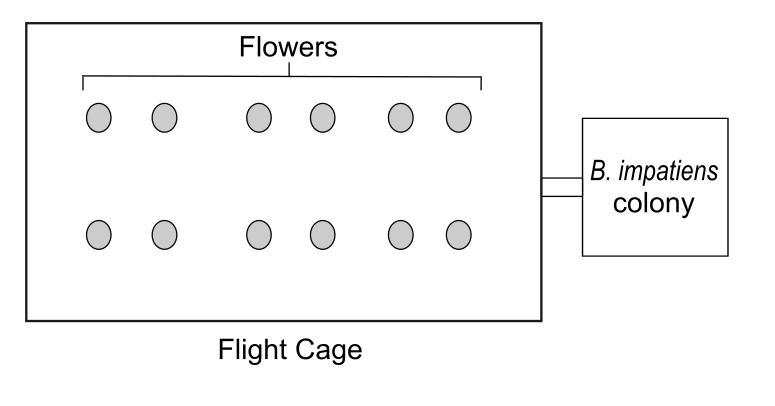
**

**Supp. Figure S2.**

Fitted results of binomial GLMM to determine effects of flower shape on the presence or absence of feces on flowers (0 indicates no feces present).


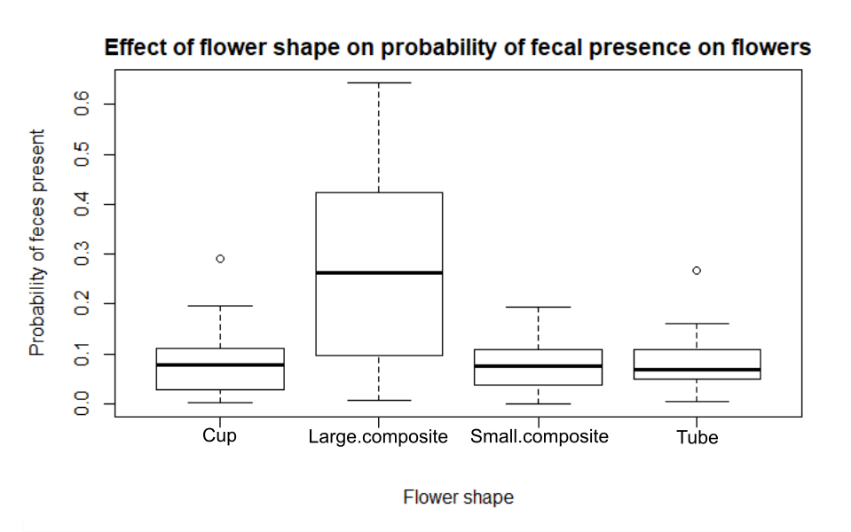


**Supp. Table S1.**

Model selection for binomial logistic regression of the presence or absence of feces on flowers only (not including disks).

| **Model** | **df** | **logLik** | **AICc** | **delta** | **weight** |
| --- | --- | --- | --- | --- | --- |
| flower.type + colony + mean.num.workers + offset(log(num.flwrs.type)) + (1 \| trial.id) | 8 | -74.97 | 166.67 | 0 | 0.56 |
| flower.type + colony + offset(log(num.flwrs.type)) + (1 \| trial.id) | 7 | -76.46 | 167.49 | 0.82 | 0.37 |
| flower.type + mean.num.workers + offset(log(num.flwrs.type)) + (1 \| trial.id) | 6 | -81.27 | 174.95 | 8.28 | 0.01 |

Variables are defined as follows: flower.type = flower shape (cup, tube, large composite, small composite), colony = which *B. impatiens* colony was used (1-3), mean.num.workers = mean number of workers (counted at 15 minute intervals) foraging in the flight cage during each trial, num.flwrs.type = number of flowers of the shape on which the feces being measured was found.

**Supp Table S2.**

Results from binomial GLMM showing how flower shape influenced the likelihood of defecation on flowers.

| **Variable** | **Estimate** | **SE** | **z value** | **P value** |
| --- | --- | --- | --- | --- |
| (Intercept)** | -8.76 | 2.00 | -4.39 | 0.0000 |
| Small composite | -0.54 | 0.83 | -0.65 | 0.5134 |
| Large composite** | 2.17 | 0.57 | 3.83 | 0.0001 |
| Tubular | -0.12 | 0.75 | -0.17 | 0.8682 |
| Colony 2** | 4.01 | 1.31 | 3.07 | 0.0022 |
| Colony 3** | 4.08 | 1.51 | 2.70 | 0.0069 |
| Mean num. of workers | 0.14 | 0.09 | 1.69 | 0.0917 |

N = 208 flowers and 31 trials; Small composite = flower shape, composite flowers with a shorter disk flower receptacle diameter than ray petal length; Large composite = flower shape, composite flowers with a longer disk flower receptacle diameter than ray petal length; Tubular = flower shape, flowers with a long tubular corolla; Colony = Unique colony ID (1-3). The symbol ** indicates *P* < 0.01.

**Supp Table S3.**

Contrasts from binomial GLMM showing pairwise comparisons of the likelihood of defecation on different flower shapes.

| **Pairwise comparisons** | **Estimate** | **SE** | ***z*** | ***P*** |
| --- | --- | --- | --- | --- |
| Cup: small composite | 0.54 | 0.83 | 0.65 | 0.91 |
| Cup: large composite*** | -2.17 | 0.57 | -3.83 | <0.001 |
| Cup: tube | 0.12 | 0.75 | 0.17 | 1.00 |
| Small composite: large composite* | -2.71 | 0.88 | -3.10 | <0.05 |
| Small composite: tube | -0.42 | 0.90 | -0.47 | 0.97 |
| Large composite: tube* | 2.29 | 0.79 | 2.91 | <0.05 |

Pairwise comparisons were performed on the log-odds ratio scale and corrected using a Tukey adjustment. The symbol * indicates *P* < 0.05 and ***indicates *P* < 0.001.
